# Tension pneumoperitoneum caused by intestinal perforation from underlying colon cancer: a case report

**DOI:** 10.1186/s13256-020-02437-2

**Published:** 2020-07-22

**Authors:** Woo Jin Joo, Yusuke Kuwahara, Yoko Asaka, Daisuke Mizu, Shigeo Hara, Koichi Ariyoshi

**Affiliations:** 1 Department of Pharmacoepidemiology, Graduate School of Medicine and Public Health, Kyoto University, Yoshidakonoe-cho, Sakyo-ku, Kyoto, 606-8501 Japan; 2 Trauma and Critical Care Center, Tokyo Metropolitan Bokutoh Hospital, 23-15 Kotobashi, 4-Chome, Sumida-ku, Tokyo, Japan; 3 Emergency Department, Kobe City Medical Center General Hospital, 2-1-1 Minatojima-minamimachi, Chuo-ku, Kobe, Hyogo Japan; 4 Department of Diagnostic Pathology, Kobe City Medical Center General Hospital, 2-1-1 Minatojima-minamimachi, Chuo-ku, Kobe, Hyogo Japan

**Keywords:** Abdominal compartment syndrome, Case report, Colon cancer, Decompression, Tension pneumoperitoneum

## Abstract

**Background:**

Tension pneumoperitoneum, a form of abdominal compartment syndrome, is an important clinical condition. Increased pressure in the intraperitoneal cavity leads to respiratory and circulatory instability. Most of the reported cases include complications due to active air infusion into the peritoneal cavity or trauma; however, few reports are available on tension pneumoperitoneum caused by perforation from colon cancer. We present a case of a patient with tension pneumoperitoneum caused by gastrointestinal perforation owing to colon cancer.

**Case presentation:**

A 63-year-old Japanese man with altered mental state was brought to our emergency department. He was in shock, and an abdominal radiograph suggested gastrointestinal perforation. Despite rapid fluid infusion and inotropic support, his condition deteriorated. His abdomen was tensely distended; abdominal computed tomography showed significant intra-abdominal gas. Following immediate needle decompression, his circulatory status improved. Emergent laparotomy revealed an approximately 10-cm tumor (adenocarcinoma) in the colon, which caused the perforation.

**Conclusions:**

A perforated wall or the surrounding omental fat that acts as a one-way valve could lead to tension pneumoperitoneum without active air infusion. Although tension pneumoperitoneum is a life-threatening condition, it is reversible if prompt diagnosis and immediate decompression are performed. Physicians should always consider this condition as one of the causes of shock or cardiopulmonary arrest, even without an apparent medical history of active air infusion or trauma.

## Background

Pneumoperitoneum is the presence of free air within the peritoneal cavity [1, 2]. It usually results from a perforation of the hollow viscus of the abdomen. Other causes include pelvic inflammatory disease, gynecologic examination procedures, asthma, barotrauma, bronchoscopy, and cardiopulmonary resuscitation [3]. While there is benign pneumoperitoneum that is due to asymptomatic free intra-abdominal air, tension pneumoperitoneum (TP) is a life-threatening form of pneumoperitoneum. TP is a condition in which the pressure of intra-abdominal free air is high enough to compromise blood flow and visceral function [1]. It is one form of abdominal compartment syndrome (ACS), which is defined as intra-abdominal pressure (IAP) > 20 mmHg that is associated with new organ dysfunction [4]. Although the threshold pressure of TP is not defined, elevated IAP compresses the inferior vena cava and decreases preload in TP. Decreased venous return leads to decreased cardiac output, which results in hypotension and systemic hypoperfusion. Moreover, the diaphragmatic excursion is inhibited, and decreased tidal volume leads to hypercapnia and respiratory acidosis. Immediate decompression is crucial to TP treatment.

The pathophysiology of TP and its treatment strategy should be widely recognized because it can be life-threatening, and adequate management is important to save patients. In this report, we present a case of a patient with TP caused by gastrointestinal perforation owing to colon cancer.

## Case presentation

**Fig. 1:**
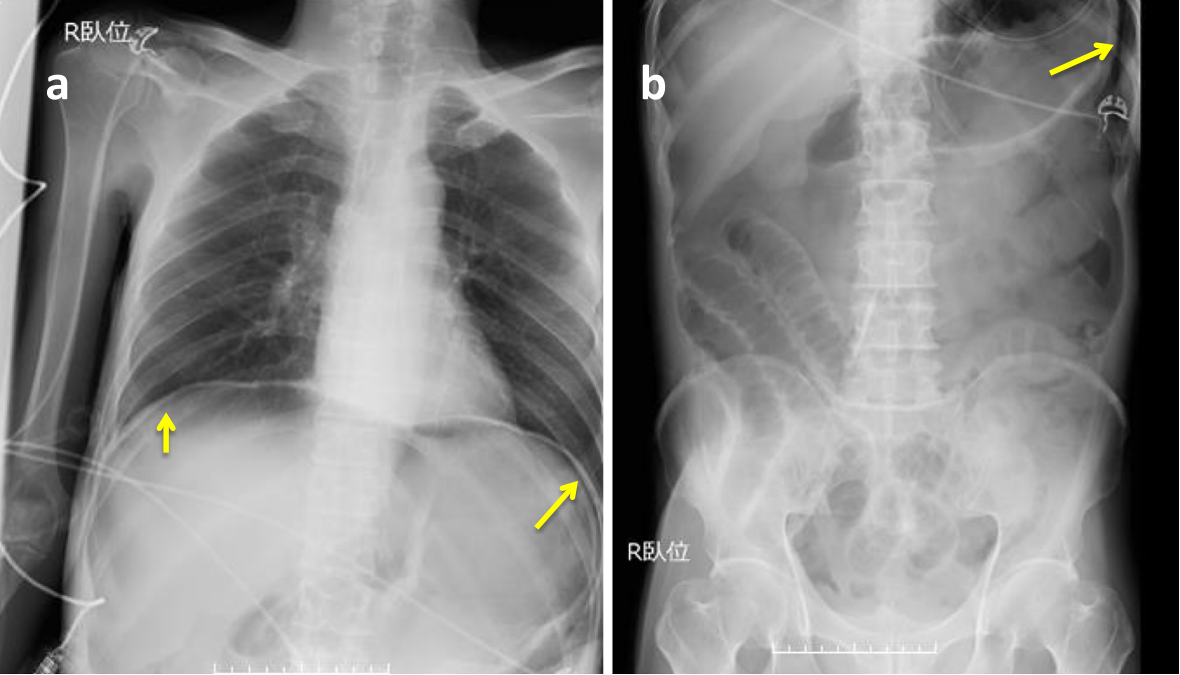
Chest (**a**) and abdominal (**b**) radiographs showing free air (*yellow arrows*)

**Fig. 2:**
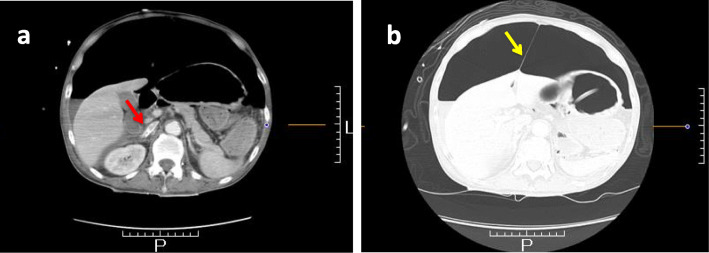
Contrast-enhanced computed tomography showing massive pneumoperitoneum. The inferior vena cava was collapsed (**a**, *red arrow*), and the round ligament of the liver was stretched owing to increased intra-abdominal pressure (**b**, *yellow arrow*)

**Fig. 3:**
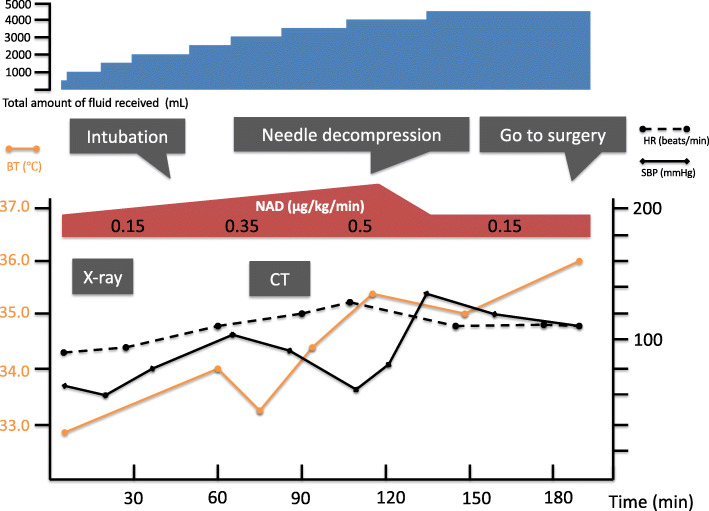
Timeline of the patient’s clinical course in the emergency department. BT, body temperature; CT, computed topography; HR, heart rate; NAD, noradrenaline; SBP, systolic blood pressure

A 63-year-old Japanese man with an altered mental state was brought to our emergency department. He had no major previous illness and was not taking any medication. His family history was unremarkable. His social history included chronic alcohol abuse, but he had no history of smoking. Two weeks prior to admission, he started developing anorexia and drank only alcohol. Although he could go out of the house until then, he gradually developed weakness. On the day of admission, his common-law wife found him unconscious, and he was not responsive; hence, he was brought to the hospital via ambulance. He had no apparent history of abdominal pain, shoulder pain, or dyspnea. His initial examination in the emergency department revealed an altered level of consciousness (Glasgow Coma Scale score, 8). His vital signs were the following: body temperature, 33.1 °C; blood pressure, 51/21 mmHg; and heart rate, 86 beats/minute. His pulse oximetry waveform was initially undetectable. His physical examination revealed the following: pale face; round and isocoric pupils (both 4 mm) that were responsive to light reflex prompt; cold extremities; and dirty appearance, especially in the intraoral cavity. His abdomen was tensely distended and tympanic. Bedside ultrasonography showed a poor image because of the large volume of abdominal air, and thus we were unable to obtain remarkable findings. Laboratory tests revealed the following: white blood cell count, 22,100/μl; hemoglobin, 15.8 g/dl; and C-reactive protein, 32.9 mg/dl (normal range, 0.00–0.50 mg/dl). Arterial blood gas analysis showed respiratory and metabolic acidosis (pH, 7.040; partial pressure of carbon dioxide in arterial blood, 64.3 mmHg; lactate, 8.7 mmol/L [normal range, 0.5–1.6 mmol/L]; bicarbonate, 12.7 mmol/L). Immediate resuscitation was provided using intravenous fluids, endotracheal intubation, a high dose of noradrenaline, and empiric antibiotics (meropenem and vancomycin). Chest and abdominal radiographs showed intraperitoneal gas (Fig. 1), and septic shock caused by gastrointestinal tract perforation was suspected. Fluid resuscitation was continued, and the noradrenaline dose was subsequently increased to 0.5 μg/kg/minute; however, his condition did not improve. Computed tomography (CT), performed during preparation for surgery, revealed a massive pneumoperitoneum that had collapsed the inferior vena cava and stretched up to the round ligament of the liver (Fig. 2). His condition deteriorated, and his abdomen was markedly distended. High airway pressures were noted, and ventilation became difficult. We initially considered sepsis, but at this point, we suspected TP as the cause of shock. Immediate needle decompression was performed using an 18-gauge cannula through the left abdominal wall. The release of gas was audible, and significant improvement in hemodynamic and respiratory status was noted. He was transferred to the operating room approximately 1 hour after needle decompression. The timeline from admission to surgery is shown in Fig. 3.

Emergent laparotomy revealed an approximately 10-cm tumor in the colon with perforation. Colectomy and ileostomy were performed. The pathology of the tumor was adenocarcinoma of the descending colon (pT3, pN0, pM0, pStage IIA). The patient underwent intensive care postoperatively and was extubated on day 4. He was discharged from the intensive care unit on day 10. He developed an intra-abdominal abscess, surgical site infection, and drug-induced acute kidney injury, which were treated for several weeks. Finally, he was transferred to another hospital for rehabilitation on day 41. He was able to walk and eat independently after rehabilitation; however, he subsequently developed peritoneal metastasis. He declined additional cancer treatment, and thus only palliative care was performed.

## Discussion and conclusions

TP is an extreme form of pneumoperitoneum, and the pathophysiology is similar to that of ACS. The abdomen is a closed anatomic space surrounded by the diaphragm and abdominal musculature; therefore, IAP increases as the intra-abdominal volume increases [5]. According to the consensus of the World Society of the Abdominal Compartment Syndrome, ACS is defined as a sustained IAP > 20 mmHg that is associated with new organ dysfunction [4]. While primary ACS is caused by injury or disease in the abdominopelvic region, secondary ACS does not originate from it. Risk factors for ACS include diminished abdominal wall compliance, increased intraluminal or intra-abdominal contents, and massive fluid resuscitation. The causes of ACS include burn, trauma, acute pancreatitis, and ruptured abdominal aortic aneurysm. TP is a form of ACS in which the IAP increases rapidly with increased intra-abdominal air volume. Similar to other types of ACS, increased IAP elevates the diaphragm, reduces lung volume, leads to ventilatory insufficiency, and decreases venous return and diastolic filling, thereby decreasing cardiac output. This manifests as shock and hypercarbic respiratory distress, which can be life-threatening if immediate abdominal decompression is not performed.

Most reported causes of TP are complications of upper or lower gastrointestinal endoscopy [6, 7] and cardiopulmonary resuscitation [8, 9]. Air insufflation by endoscopy causes a large amount of gas leakage into the peritoneal cavity through the perforated wall of the gastrointestinal tract owing to an iatrogenic procedure or ulcer. Improper intubation (i.e., intubation to the esophagus) or gastric rupture due to chest compression during cardiopulmonary resuscitation can result in TP [8]. Other reported causes include trauma [10], scuba diving [11], mechanical ventilation [12], obstipation [3], and unhealed abdominal mesh [13]. Regardless of the cause, a large amount of gas infused into the peritoneal cavity results in acute ACS.

In our patient’s case, a relatively common condition of gastrointestinal tract perforation due to colon cancer resulted in TP. It is unclear why TP occurred without active air infusion. A perforated wall or the surrounding omental fat might act as a one-way valve as well as in the cases of TP caused by obstipation or unhealed abdominal mesh [3, 13]. Additionally, his altered mental status resulted in progression until the leaked gas reached a high pressure in the peritoneal cavity, and TP might have been already present during admission. Because he had no medical history suggesting infusion of a large amount of gas into the gastrointestinal tract or peritoneal cavity, we did not include TP in the differential diagnosis. We initially suspected septic shock, and needle decompression was delayed. It took some time to refer the patient to a surgeon, prepare an operating room, and find anesthesiologists. The patient was admitted during daytime on a weekday, when there was another emergent surgery, thus further delaying the surgery. If TP had not been recognized and needle decompression had been delayed further, he might have experienced cardiac arrest by the time of consultation with the surgeon and preparation for surgery.

Physical examination and investigation of medical history are crucial for diagnosing TP. Physical examination in TP usually shows tachypnea with accessory muscle use, abdominal tenderness, and abdominal distension with tympanitic sound on percussion. Patients with severe circulatory failure may have tachycardia, decreased blood pressure, and loss of peripheral pulses. Laboratory tests may show hypercapnia, hypoxemia, and lactic acidosis. Medical history in TP typically includes a history of endoscopic procedure, trauma, or mechanical ventilation. However, even without such apparent medical history, it is important to consider this condition as one of the differential diagnoses of shock, respiratory distress, and cardiac arrest, because it might lead to death if intervention is delayed.

We were able to perform needle decompression using abdominal CT imaging safely. However, CT evaluation in an emergency is not mandatory, because it may lead to delayed intervention. Lateral radiographic views might help in the diagnosis and safer puncture [3].

This case suggests that perforation by colon cancer without active air infusion can lead to TP. However, not all gastrointestinal perforations lead to TP. Further studies are needed to investigate the incidence and mechanism of gastrointestinal perforations leading to TP.

## Data Availability

Not applicable.

## Abbreviations

ACS: Abdominal compartment syndrome
CT: Computed tomography
IAP: Intra-abdominal pressure
TP: Tension pneumoperitoneum
